# Melatonin mediates phenolic acids accumulation in barley sprouts under MeJA stress

**DOI:** 10.3389/fnut.2024.1403293

**Published:** 2024-06-04

**Authors:** Xin Tian, Renjiao Zhang, Zhengfei Yang, Jiangyu Zhu, Weiming Fang, Runqiang Yang, Yongqi Yin

**Affiliations:** ^1^College of Food Science and Engineering, Yangzhou University, Yangzhou, Jiangsu, China; ^2^College of Food Science and Technology, Nanjing Agricultural University, Nanjing, Jiangsu, China

**Keywords:** phenolic acids, barley sprouts, methyl jasmonate, melatonin, gene expression

## Abstract

Phenolic acids are secondary metabolites in higher plants, with antioxidant, anticancer, and anti-aging effects on the human body. Therefore, foods rich in phenolic acids are popular. Methyl jasmonate (MeJA) promoted phenolic acids accumulation but also inhibited sprout growth. Melatonin (MT) was a new type of plant hormone that not only alleviated plants’ abiotic stress, but also promoted the synthesis of plant-stimulating metabolism. This study aimed to elucidate the mechanism of exogenous MT on the growth and development, and phenolic acids metabolism of barley sprouts under MeJA treatment. The results showed that MT increased the phenolic acids content in sprouts by increasing the activities of phenylalanine ammonia-lyase and cinnamic acid 4-hydroxylase, and up-regulating the gene expression of *phenylalanine ammonia-lyase*, *cinnamic acid 4-hydroxylase*, *4-coumarate: coenzyme a ligase*, and *ferulic acid-5-hydroxylase*. MT attenuated the growth inhibition of barley sprouts under MeJA stress by increasing the activities of regulated antioxidant enzymes and the expression of their corresponding genes. Furthermore, MT increased the NO content and induced Ca^2+^ burst in barley sprouts under MeJA stress. These events were inhibited by DL-4-Chlorophenylalanine. These results suggested that MT ameliorated growth inhibition and promoted the biosynthesis of phenolic acids in barley sprouts under MeJA stress.

## 1 Introduction

Phenolic acids are secondary metabolites found in higher plants, primarily synthesized through the phenylpropane metabolic pathway (1). Studies indicated that phenolic acids play a significant role in plant defense systems, by limiting the generation of free radicals, reducing levels of reactive oxygen species, and enhancing antioxidant capacity (2, 3). In addition, in the human body, phenolic acids also exhibit strong antioxidant properties (4–6), effectively eliminating free radicals generated, delaying cell aging, inhibiting tumor growth, and promoting metabolism. However, phenolic acids are unable to be produced within the human body and must be obtained through external sources (7). Therefore, increasing the content of phenolic acids in plant-based foods has garnered widespread attention.

In the past few years, extensive studies have been conducted on the metabolism and accumulation of phenolic acids (6, 8). Research has found that germination under abiotic stress conditions can significantly increase the content of phenolic acids in barley (9). Methyl jasmonate (MeJA), as a plant hormone, plays various roles in plant growth metabolism, including protein synthesis, enzyme metabolism, and photosynthesis (10–12). Studies have shown that MeJA treatment can significantly increase the content of secondary metabolites in plants, especially phenolic compounds. Research by Rahmati et al. (13) found that treatment with 25 μM MeJA significantly increased the total phenolics content in *Carum carvi* L. Palai et al. (14) discovered that treatment with 10 μM MeJA could significantly increase the content of flavonoid compounds in grapes. In the preliminary experiments, it was found that 100 μM MeJA significantly increased phenolic acids content in barley sprouts. However, it was also discovered that MeJA treatment could inhibit sprouts’ growth and development. Therefore, it is necessary to seek a method to alleviate growth inhibition and further promote the synthesis of phenolic acids on barley sprouts under MeJA stress.

Melatonin (MT) was initially discovered in plants in 1995 (15). MT in plant cells has various physiological effects (16, 17), including regulating plant growth, seed germination, and enhancing the photosynthetic rate of plants. Furthermore, research has indicated MT could alleviate plant stress by improving oxidative-reductive homeostasis (18), cell membrane stability (19), and photosynthetic efficiency (18). The research also found that exogenous MT serves as a stimulant for secondary metabolites in many plants. For example, it increased the synthesis of phenolic acids in tomatoes (20), citrus fruits (21), and cowpeas (22). Recent studies (23) have shown that the synergistic effect of *Pseudomonas fluorescens* and MT improved salt tolerance, and further increased the production of secondary metabolites in mustard sprouts under biotic stress. Similarly, in our previous studies (24, 25), it was demonstrated that MT could improve the salt tolerance of soybeans and barley sprouts, remove growth inhibition, and increase the content of isoflavones in soybean sprouts and phenolic acids in barley sprouts. These studies suggested that MT may be a promising plant hormone that can enhance biosynthesis, and maintain biomass under stress. However, current research has not yet found how MT alleviates the stress of MeJA on barley sprouts and further promotes phenolic acids synthesis.

In this study, the mechanism of exogenous MT on the development and total phenolic acids metabolism of barley sprouts under MeJA stress from physiological metabolism and gene transcription perspectives were investigated. The study objective was to elucidate the role of MT in enriching total phenolic acids in barley sprouts and unravel the underlying multifaceted mechanisms. This study served to lay the groundwork for further exploration into the synthesis and regulation mechanisms of phenolic acids in barley sprouts, offered resource reserve, and a theoretical basis for targeted plant-based foods.

## 2 Materials and methods

### 2.1 Materials and reagents

Barley seeds (Su Hullless 2), supplied by Jiangsu Academy of Agricultural Sciences (Nanjing, China), were stored at −20°C. MT, MeJA, and DL-4-Chlorophenylalanine (MT inhibitor, PCPA) were bought from Sigma (Shanghai, China), and the remaining reagents were purchased from Macklin (Shanghai, China).

### 2.2 Experimental design and treatment

Barley seeds were selected, sterilized in sodium hypochlorite solution, and subsequently rinsed with deionized water to pH = 7.0. Soaked for 6 h at 25°C in deionized water. The soaked barley was placed in individual germination trays (for a single treatment group) and germinated for 6 days at 25°C in the dark. Each treatment was sprayed with 40 mL of a different solution every 12 h during germination. (1) M: sprayed with 100 μM MeJA; (2) MM: sprayed with a mixture of 100 μM MeJA and 1 mM MT (v:v = 1:1); (3) MP: sprayed with a mixture of 100 μM MeJA and 100 μM PCPA (v:v = 1:1); (4) MMP: sprayed with a mixture of 100 μM MeJA, 1 mM MT and 100 μM PCPA (v:v:v = 1:1:1). On the 4th and 6th day, the barley sprouts were collected at random, stored in a − 20°C refrigerator, and kept for measurement of relevant indicators.

### 2.3 Sprout length and fresh weight

Determination of sprout length and fresh weight (FW) according to Yin et al. (24) method.

### 2.4 Free amino acids and soluble protein content

The content of free amino acids in barley sprouts was determined by the method of Ma et al. (26). In detail, 1.0 g of barley sprouts were ground with 10% acetic acid to obtain a uniform slurry, then centrifuged to collect the supernatant. The supernatant was mixed with ninhydrin and vitamin C, heated for 15 min, and measured at the wavelength of 570 nm.

Determination of soluble proteins with the Assay Kit (A045-2-1, Nanjing Jiancheng Bioengineering Institute, China).

### 2.5 Malondialdehyde, hydrogen peroxide, and superoxide anion content

According to the method of Zhuang et al. (27) determined the malondialdehyde (MDA) and superoxide anion ( $O_{2}^{–•}$ ) content. Barley sprouts were ground with trichloroacetic acid. After centrifugation, the supernatant was boiled with thiobarbituric acid for 20 min. The absorbance values of the supernatant were determined at 450 nm, 532 nm, and 600 nm, respectively.

The production of $O_{2}^{–•}$ was determined by monitoring hydroxylamine nitrate in the presence of $O_{2}^{–•}$ generators. Barley sprouts were ground to a homogenate with 65 mM phosphate buffer and centrifuged at 8,000 × *g* for 10 min. The 1 mL supernatant was mixed with 0.9 mL of 65 mM phosphate buffer (pH = 7.8) and 0.1 mL of 10 mM hydroxylammonium chloride. After incubation at 25°C for 20 min, 17 mM sulfanilic acid and 7 mM α-naphthylamine were added to the mixture, and the absorbance was measured at 530 nm after holding at 25°C for 20 min. The standard curve of sodium nitrate was used to calculate the rate of $O_{2}^{–•}$ production.

Hydrogen peroxide (H_2_O_2_) content was determined using Assay Kit (A064-1-1, Nanjing Jiancheng Bioengineering Institute, China).

### 2.6 Total phenolics, total phenolic acids, and nitric oxide content

Total phenolics and total phenolic acids contents were determined with reference to Ma et al. (26). Specifically, barley seedling was homogenized with 50% methanol to obtain a homogenate. The homogenate was centrifuged to obtain the supernatant. The supernatant was thoroughly mixed with Folin-phenolic and sodium carbonate, then incubated at 25°C for 2 hours in the dark. The absorbance value was measured at 765 nm, and the total phenolics content was calculated based on the gallic acid standard curve.

Furthermore, the supernatant was mixed with 50% methanol, 0.3% sodium dodecyl sulfate, and 0.6% ferric chloride, then mixed thoroughly and placed in the dark for 5 min. The absorbance at 760 nm was read to determine the total phenolic acids content.

Nitric oxide (NO) content was determined using Assay Kit (XZK-15995, Zexuankang Bioengineering Institute, China).

### 2.7 Intracellular free calcium, H_2_O_2_, and $O_{2}^{–•}$

The intracellular free calcium, H_2_O_2_, and $O_{2}^{–•}$ fuorescence staining according to the method of Yin et al. (24). Germinated barley seedlings at 4 days under different treatments were collected. The root tips were cut to a length of 3 mm. The root tips were incubated in Hanks balanced salt buffer solution (HBSS) containing fluorescent dyes (Fluo-4 AM, dihydroethidium, and 2′,7′-dichlorofluorescein diacetate), and stained at 4°C for 2 h. After staining, the root tips were washed thrice with HBSS and then incubated in the dark at 25°C for 2 h. Subsequently, images were captured using an LSM 880 NLO confocal laser scanning microscope (Leica Excitation Technology Co., Ltd., Germany), with an excitation wavelength of 488 nm and an emission wavelength of 520 nm.

### 2.8 Antioxidant enzyme activity

The activities determination of peroxidase (POD) and superoxide dismutase (SOD) were conducted following the method of Wang et al. (28). Barley sprouts were ground in an ice bath containing sodium phosphoric acid buffer (pH = 7.0, 50 mM). The suspensions were centrifuged, and the supernatant was used to measure the enzyme activity. A change of 0.01 at OD_470 nm_ per minute was expressed as one unit of POD activity. A change of 0.01 at OD_560 nm_ per minute was expressed as one unit of SOD activity.

### 2.9 2,2′-azino-bis (3-ethylbenzothiazoline-6-sulfonic acid) and 2,2-diphenyl-1-picrylhydrazyl radical scavenging assay

Following the methods described in references (29), measure the 2,2′-azino-bis (3-ethylbenzothiazoline-6-sulfonic acid) (ABTS) and *2,2-diphenyl-1-picrylhydrazyl* (DPPH) free radical scavenging rates in the barley. Absorbance at 734 nm was read on a spectrophotometer to determine ABTS free radical scavenging activity. DPPH scavenging was calculated by measuring absorbance at 515 nm using vitamin C as a positive control.

### 2.10 Phenylalanine ammonia-lyase and cinnamic acid 4-hydroxylase activities

The activities of Phenylalanine ammonia-lyase (PAL) and cinnamic acid 4-hydroxylase (C4H) were determined by the method of Yin et al. (24). Barley sprouts and phosphate buffer were homogenized at low temperatures. The supernatant was centrifuged, and the absorbance of the supernatant and reaction mixture was measured at wavelengths 290 nm and 345 nm, respectively. A change of 0.01 per minute is one unit of enzyme activity.

### 2.11 Gene expression

Barley sprouts total RNA was isolated employing the Takara MiniBEST Plant RNA Extraction Kit (9,769, Takara, China), followed by cDNA synthesis using the PrimeScriptH RT Kit (RR092S, Takara, China). The study utilized sequence-specific primers in Table 1 for quantitative real-time PCR (qRT-PCR) analysis. Each treatment was assessed three times by qRT-PCR using the ABI 7500 Sequence Detection System (Foster City, CA, USA) and SYBR® Pre-mix Ex TaqTM (RR820A, Takara, China). Relative gene expression levels were calculated using the 2-^ΔΔCt^ method.

**Table 1:** Sequence-specific primers used in the present study.

Gene name	Forward primer (5′–3′)	Reverse primer (5′–3′)
*HvPAL*	CACTGAATGCCGATCATACCC	CCGTTCCAACCCTTGAGACA
*HvC4H*	AGCTCGCCGCCTACAACATC	GCCTCCACGTGCTTCTCCTC
*Hv4CL*	GGTGGAGATCGCCAAGAGCC	CTCCGTCATCCCGTACCCCT
*HvC3H*	ATCACCGCTGGGATGGACA	GGTACGGCAGGTTCTGGAAGT
*HvCOMT*	GCCGTCAAAGGCATCAACT	GCAAGGCGTCATAGCAGTTC
*HvF5H*	TCGACGACATGCTCGCCTTC	TCCCGCCAAACATCACGTCC
*HvPOD*	ACTGTTTCGGTCCAAGGCTG	TCTTGATGCTGTCGATGACG
*HvSOD*	CCCCTCACCAAGTCAGTCAT	ATTGCAAGTCGGTGTCCTTC
*HvActin*	TCGTGAGAAGATGACCCAGA	CCGAGTCCAGCACAATACCT

### 2.12 Statistical analysis

The outcomes were presented as the mean ± standard deviation (SD) of duplicated determinations. Statistical analysis was performed using SPSS 18.0 software. Duncan’s multiple range test, with a significance level of 0.05 (*p* < 0.05), was employed to evaluate significant variances.

## 3 Results

### 3.1 Morphology, length, weight, free amino acid, and soluble protein content

**Figure 1:**
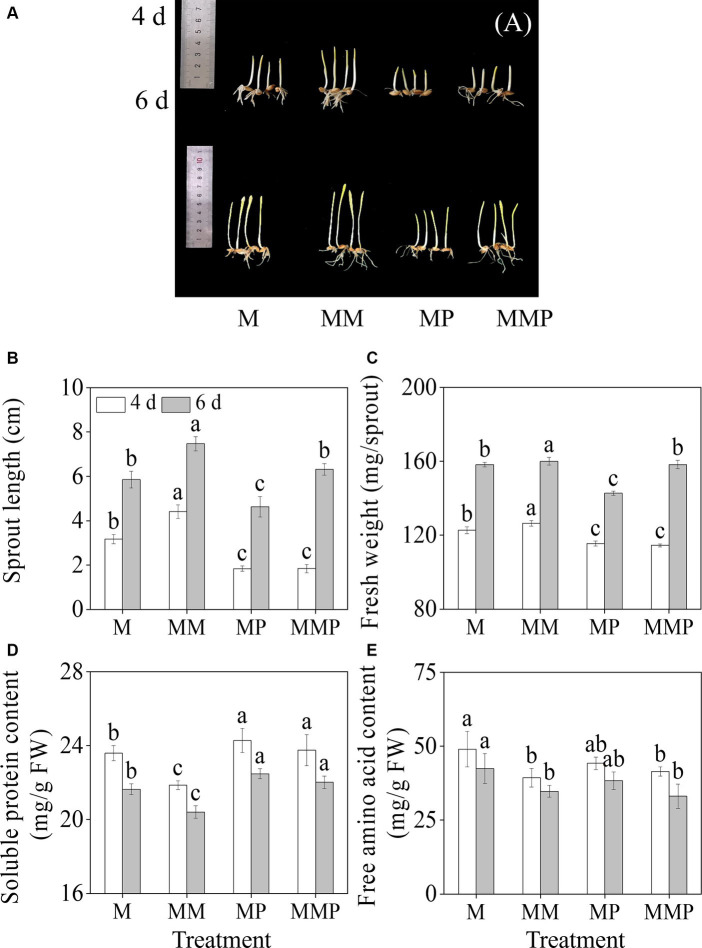
The effects of MT on growth performance**(A)**, sprout length **(B)**, fresh weight **(C)**, free amino acid content **(D)**, and soluble protein content **(E)** in barley sprouts under MeJA stress. Error bars indicate the standard deviations of each data point (*n* = 3). Lowercase letters indicate significant (*p* < 0.05) differences between treatments for the same germination time. M: 100 μM MeJA, MM: 100 μM MeJA plus 0.1 mM MT, MP: 100 μM MeJA plus 100 μM PCPA, MMP: 100 μM MeJA plus 0.1 mM MT plus 100 μM PCPA. The same as below.

Compared with MeJA treatment, exogenous MT significantly increased (Figures 1A,B, *p* < 0.05) the length of the sprout, and the length increased by 41.9 and 27.6%, respectively. MT notably increased the fresh weight of sprouts (Figure 1C, *p* < 0.05). However, the PCPA (MT inhibitor) significantly reduced the fresh weight and sprout length of four-day-old sprouts, whether under MeJA treatment or MeJA plus MT treatment. Furthermore, compared with MeJA treatment, the addition of exogenous MT resulted in a substantial reduction in the content of soluble proteins and free amino acids present in the sprouts (*p* < 0.05, Figures 1D,E), with decreased by of 8.0 and 20.4%, respectively, at 4-day-old. Additionally, the addition of PCPA significantly increased the content of soluble proteins in sprouts under MeJA and MeJA plus MT treatments.

### 3.2 Total phenolics, total phenolic acids, and no content

**Figure 2:**
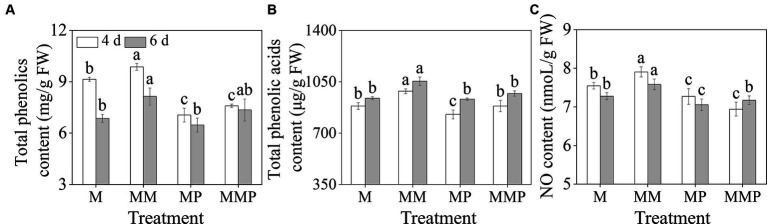
The effects of MT on total phenolics content **(A)**, total phenolic acids content **(B)**, and NO content **(C)** in barley sprouts under MeJA stress.

Compared with MeJA treatment, the addition of MT significantly increased the total phenolics, total phenolic acids, and NO content in sprouts (Figure 2, *p* < 0.05). The total phenolics and NO content reached their maximum values on the 4th day (Figures 2A,B), which were 9.86 mg/g FW and 7.91 nmoL/g FW, respectively. The total phenolic acids content reached maximum value on the 6th day, which was 1054.08 μg/g FW. Additionally, PCPA significantly reduced the total phenolics, total phenolic acids, and NO content on the 6th day, regardless of MeJA treatment or MeJA plus MT treatment. These results suggested that NO, as a signaling molecule, mediated the promotion of phenolic acids under MT plus MeJA treatment.

### 3.3 Malondialdehyde, hydrogen peroxide, and superoxide anion content

**Figure 3:**
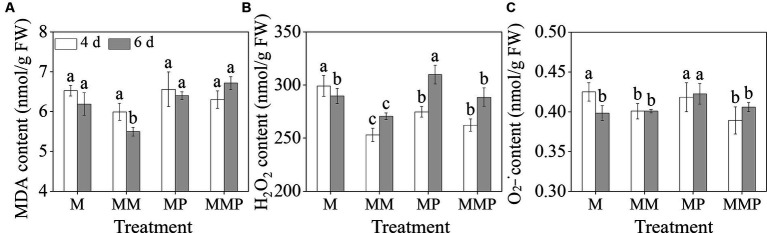
The effects of exogenous MT on MDA content **(A)**, H_2_O_2_ content **(B)**, and $O_{2}^{–•}$ content **(C)** in barley sprout under MeJA stress.

The exogenous MT significantly reduced membrane damage and reactive oxygen species (ROS) content in barley sprouts under MeJA treatment (Figure 3, *p* < 0.05). Specifically, MT decreased the H_2_O_2_, and $O_{2}^{–•}$ content in 4-day-old barley sprouts by 16.1 and 5.7%, respectively, and also significantly reduced (Figures 3B,C, *p* < 0.05) the MDA and H_2_O_2_ content in 6-day-old sprouts by 13.0 and 5.5% (Figures 3A,B, *p* < 0.05), respectively, under MeJA treatment. Furthermore, the addition of PCPA significantly increased the MDA and H_2_O_2_ content in 6-day-old sprouts under MeJA combined with MT. These results indicated that MT can serve as an antioxidant in barley sprouts under MeJA stress.

### 3.4 Intracellular free calcium, H_2_O_2_, and $O_{2}^{–•}$ fluorescence

The root tips of all experimental samples were subjected to staining with fluorescence for free calcium, H_2_O_2_, and $O_{2}^{–•}$ . The MT plus MeJA treatment exhibited the highest intracellular free calcium (green) fluorescence intensity, compared to the other treatments. The application of MT reduced the fluorescence intensity of H_2_O_2_ (red) and $O_{2}^{–•}$ (blue) at the root tips compared to MeJA stress. These findings suggested that the accumulation of ROS was alleviated under the MT plus MeJA treatment.

### 3.5 Antioxidant enzyme system

**Figure 4:**
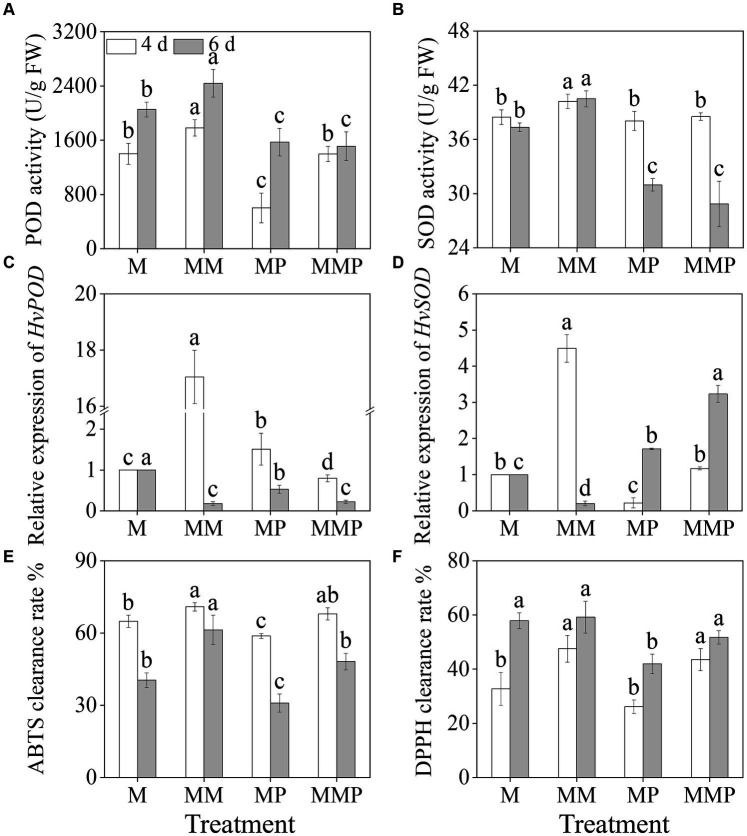
Effects of MT on the activities of POD **(A)** and SOD **(B)**, and the gene expression levels of *HvPOD* **(C)** and *HvSOD* **(D)** in barley sprouts under MeJA stress. The clearance rate ABTS **(E)** and DPPH **(F)** of barley sprouts were measured.

The addition of exogenous MT significantly increased (Figures 4A,B, *p* < 0.05) the activities of POD and SOD in sprouts under MeJA treatment. The activities of POD and SOD in 6-day-old sprouts reached maximum values, at 1782.5 U/g and 40.2 U/g, respectively. On the 4th day, compared to MeJA treatment, the addition of MT significantly up-regulated (Figures 4C,D, *p* < 0.05) the expression levels of *HvPOD* and *HvSOD*, increased by 44.74- and 4.42-time, respectively. PCPA significantly reduced (Figures 4A,B, *p* < 0.05) the activities of POD and SOD in four-day-old sprouts under MM treatment, as well as the expression of *HvPOD* and *HvSOD* levels were down-regulated. Furthermore, MT not only significantly increased the clearance rates of DPPH and ABTS (Figures 4E,F, *p* < 0.05) under MeJA treatment, but also increased the clearance rates of DPPH and ABTS in sprouts under MeJA plus PCPA treatment. These results indicated that exogenous MT increased the clearance rate of free radicals by enhancing the antioxidant system.

### 3.6 Phenolic acids metabolizing system

**Figure 5:**
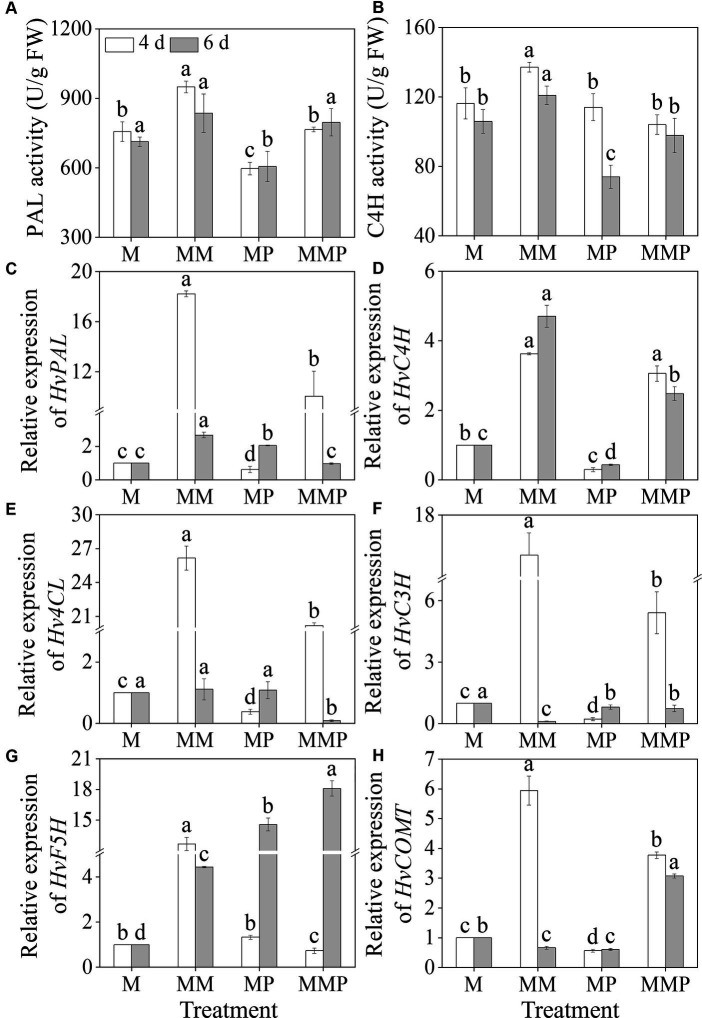
Effects of MT on the activities of PAL **(A)** and C4H **(B)**, and the gene expression levels of *HvPAL* **(C)**, *HvC4H* **(D)**, *Hv4CL* **(E)**, *HvC3H* **(F)**, *HvF5H* **(G)**, and *HvCOMT* **(H)** in barley sprouts under MeJA stress.

MT significantly increased barley sprouts’ PAL and C4H activities under MeJA treatment (Figures 5A,B, *p* < 0.05). Specifically, on the 4th day, PAL and C4H activities, under MT plus MeJA treatment, reached maximum values, at 949.35 U/g and 137.9 U/g, respectively, which were 1.3- and 1.2-fold higher than the MeJA treatment. The addition of PCPA significantly reduced the PAL and C4H activities in four-day-old sprouts under MM treatment and the C4H activity in six-day-old sprouts. Compared to MeJA treatment, the MT significantly up-regulated the gene expression levels of *HvPAL*, *HvC4H*, *4-coumarate: coenzyme a ligase* (*Hv4CL*), and *ferulic acid-5-hydroxylase* (*HvF5H*) (Figures 5C–E, G, *p* < 0.05). Specifically, at the 4th of sprouts under MM treatment, the expression levels were 18.2-, 3.6-, 26.2-, and 12.7-fold higher than the MeJA treatment. At 6th of sprouts under MM treatment, the expression levels were 2.7-, 4.7-, 1.1-, and 4.4-fold higher than MeJA treatment. Similarly, the exogenous MT significantly increased the expression levels of *HvC4H* and *caffeic acid O-methyltransferase* (*HvCOMT*) in sprouts under MeJA combined with PCPA treatment (Figures 5D,H, *p* < 0.05). These results suggested that exogenous MT promoted total phenolic acids synthesis by upregulating gene expression levels.

## 4 Discussion

Sprouted barley, as a new form of food, possesses a nutritional value far superior to that of barley itself. In recent years, with the pursuit of healthy diets, barley has been widely used in breakfast products, salads, soups, and baked goods due to its unique nutritional value and flavor (30, 31). However, how to further improve the nutritional of barley, especially to increase the total phenolic acids content, has been the direction of researchers’ efforts. Studies have shown that germination under abiotic stress increases the content of secondary metabolites in the plant (9). In this study, it was found that MT combined with MeJA treatment significantly increased the total phenolic acids content in barley sprouts (Figure 2B). This study will increase the nutritional value and health functions of existing barley sprout-related products such as barley green juice and barley sprouts powder. Based on this study, barley sprout products with richer nutrients and better health functions can be developed to meet consumer demand for health, flavor, and nutrition.

MeJA, as a phytohormone, promotes total phenolic acids content in plants (13), but reduces sprouts length and fresh weight, induces the production of ROS. In this study, it was found that the addition of 0.1 mM exogenous MT under MeJA treatment increased the shoot length and fresh weight (Figures 1B,C), decreased MDA, H_2_O_2_, and $O_{2}^{–•}$ content (Figures 3A–C). Conversely, the addition of MT inhibitor PCPA resulted in more severe oxidative damage to the sprouts, leading to a significant decrease in shoot length and fresh weight. However, the addition of MT could reverse the adverse effects caused by PCPA, and protect the integrity of the cell membrane structure. As a new type of plant hormone, MT not only has the function of alleviating damage caused by various abiotic stresses to plants (18, 19), but also can induce the production of some secondary metabolites (20, 21), including total phenols, total phenolic acids, and flavonoids. In this study, the addition of MT significantly increased the content of total phenolics and total phenolic acids (Figures 2A,B) in sprouts treated with MeJA. These results suggested that MT could act not only as an effective plant antioxidant to alleviate oxidative damage to barley sprouts caused by MeJA, but also further increase the content of phenolic acids under MeJA treatment. This research will provide a new perspective for enriching barley phenolic acids and enhancing the development of plant-based foods.

**Figure 6:**
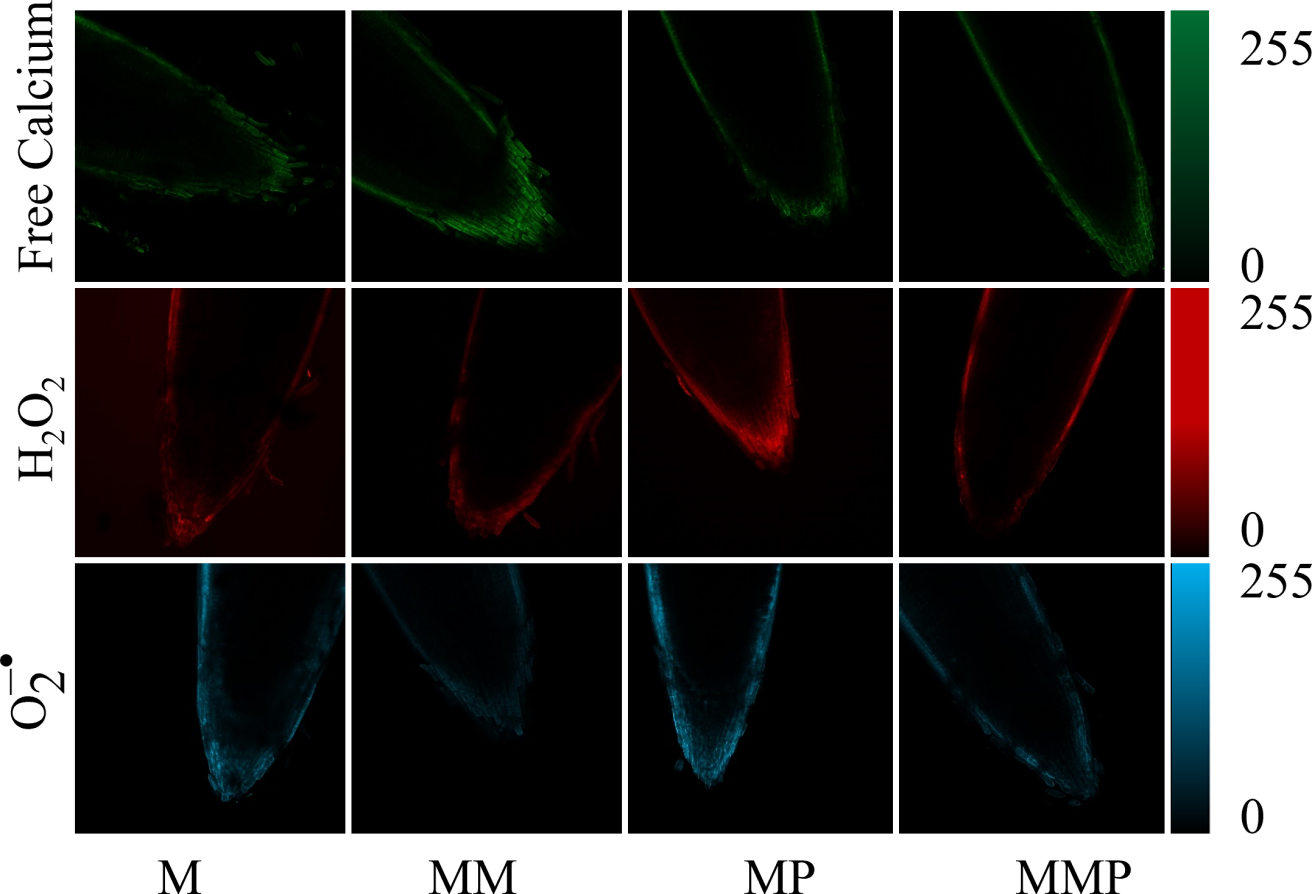
The effect of MT on root tip staining of barley sprouts under MeJA stress on the 4th day. The horizontal axis is treatment, the vertical axis is intracellular free calcium (green), H_2_O_2_ (red), and $O_{2}^{–•}$ (blue), and the scale length is 100 μm.

MT can uphold cellular osmotic equilibrium by suppressing the content of H_2_O_2_, and $O_{2}^{–•}$ , while diminishing lipid peroxidation of the cell membrane, consequently mitigating the adverse impacts of abiotic stress on plant physiology (32). Our study demonstrated that the exogenous MT significantly reduces the accumulation of H_2_O_2_ and MDA under MeJA treatment (Figures 3A–C, *p* < 0.05), enhances the ability to scavenge DPPH and ABTS free radicals (Figures 4E,F, *p* < 0.05), as well as the fluorescence intensity of H_2_O_2_, and $O_{2}^{–•}$ (Figure 6). Similar results were reported in soybean (33) and barley (24) sprouts. Under stress, plants experience an overabundance of ROS, leading to oxidative harm and the initiation of antioxidative protection mechanisms. In the realm of plant stress responses, the enzymes, POD and SOD, play essential roles. Our investigation revealed that MT effectively preserved the integrity of cell membranes by mitigating the buildup of ROS within cells, achieved through the enhancement activities of POD and SOD. Studies have shown that the decrease in H_2_O_2_, and $O_{2}^{–•}$ content is positively correlated with POD, SOD, and CAT activity and gene expression level (34). In addition, some studies (35, 36) have shown that the application of MT up-regulated the gene expression levels of *POD*, *CAT*, and *APX*, in plants under abiotic stress. Similarly, in our study, the exogenous MT significantly up-regulated the expression levels of *HvPOD* and *HvSOD* in sprouts under MeJA treatment (Figures 4C,D). Conversely, the addition of PCPA reversed the above results. These findings suggested that MT enhanced the antioxidant properties of barley sprouts by increasing POD and SOD enzyme activities and up-regulating related gene expression.

Both NO and Ca^2+^ are the signaling in plant cells (37, 38). NO could induce the opening of plasma membrane Ca^2+^ channels, increased cytoplasmic Ca^2+^ concentration, and the transmission of external information (39). The elevation of Ca^2+^ levels stimulated the production of NO, triggered programmed cell death in the infected area, and limited pathogen growth (40, 41). Additionally, the study has shown (42) that transient increases in Ca^2+^ concentration induced by oligosaccharides of galacturonic acid in *Arabidopsis thaliana* sprouts induced the expression of genes associated with phenolic compound synthesis, including *AoCHS*, *AoGST*, *AoPAL*, and *AoPR-1*. Our research has revealed that under MeJA treatment, the addition of exogenous MT significantly enhanced the Ca^2+^ fluorescence intensity and NO content in sprout root tips. Furthermore, the addition of exogenous MT upregulated the expression levels of *HvPAL*, *HvC4H*, and *Hv4CL* and increased in total phenolic content. In future studies, we will further strengthen the research on the interaction between Ca^2+^ and NO, providing a theoretical basis for exploring the alleviation of MeJA stress by MT and promoting phenolic acids accumulation.

In higher plants, the synthesis of phenolic acids is mainly through the phenylpropane metabolic pathway, where acids such as phenolic acids are produced under the action of enzymes like PAL and C4H (43, 44). The investigation demonstrated that upon exposure to MeJA, the application of exogenous MT notably enhanced the PAL and C4H activities (Figures 4A,B). Conversely, the introduction of PCPA effectively suppressed the PAL and C4H activities, suggested the participation of MT in modulating crucial enzyme functions in phenolic acids synthesis in barley sprouts subjected to MeJA-induced stress. Exogenous MT significantly increased the total phenolic and phenolic acids contents in barley sprouts (Figures 2A,B). PCPA inhibited the synthesis of total phenolics and phenolic acids in four-day-old sprouts, while MT treatment reversed the inhibitory effect of PCPA. This outcome is positively correlated with changes in the enzyme activities and contents of phenolic acids in barley sprouts, possibly due to the introduction of MT under MeJA stress mediating the synthesis of phenolic acids, thereby enhancing the key enzyme activity involved in soybean sprout phenolic substance synthesis and promoting phenolic substance synthesis. Conversely, PCPA treatment can inhibit MT synthesis, reducing phenolic substance content. The addition of MT after PCPA alleviates the inhibition, restoring some enzyme activity and content levels involved in phenolic substance synthesis. These results indicated that MT positively regulates phenolic acids synthesis in barley sprouts under MeJA stress. Numerous research studies (45, 46) have indicated that the upregulation expression levels of *PAL*, *C4H*, and *4CL* could contributes to the elevation of phenolic levels. In this study, it was observed that the MT not only up-regulated the expression levels of *HvPAL*, *HvC4H*, and *Hv4CL* but also up-regulated the expression levels of *HvF5H*. However, this is in contrast to previous findings (24), that exogenous MT promoted phenolic acids enrichment and inhibited *HvF5H* expression in sprouts under NaCl-induced stress. Our analysis suggested that the underlying cause of this discrepancy may stem from variations in gene transcription triggered by differences in stress factors or the spatiotemporal dynamics of protein translation. These results revealed that MT plus MeJA had a positive impact on the functions and levels of key enzymes involved in the phenylpropane metabolic pathway in barley, thereby increasing the phenolic acids content.

## 5 Conclusion

**Figure 7:**
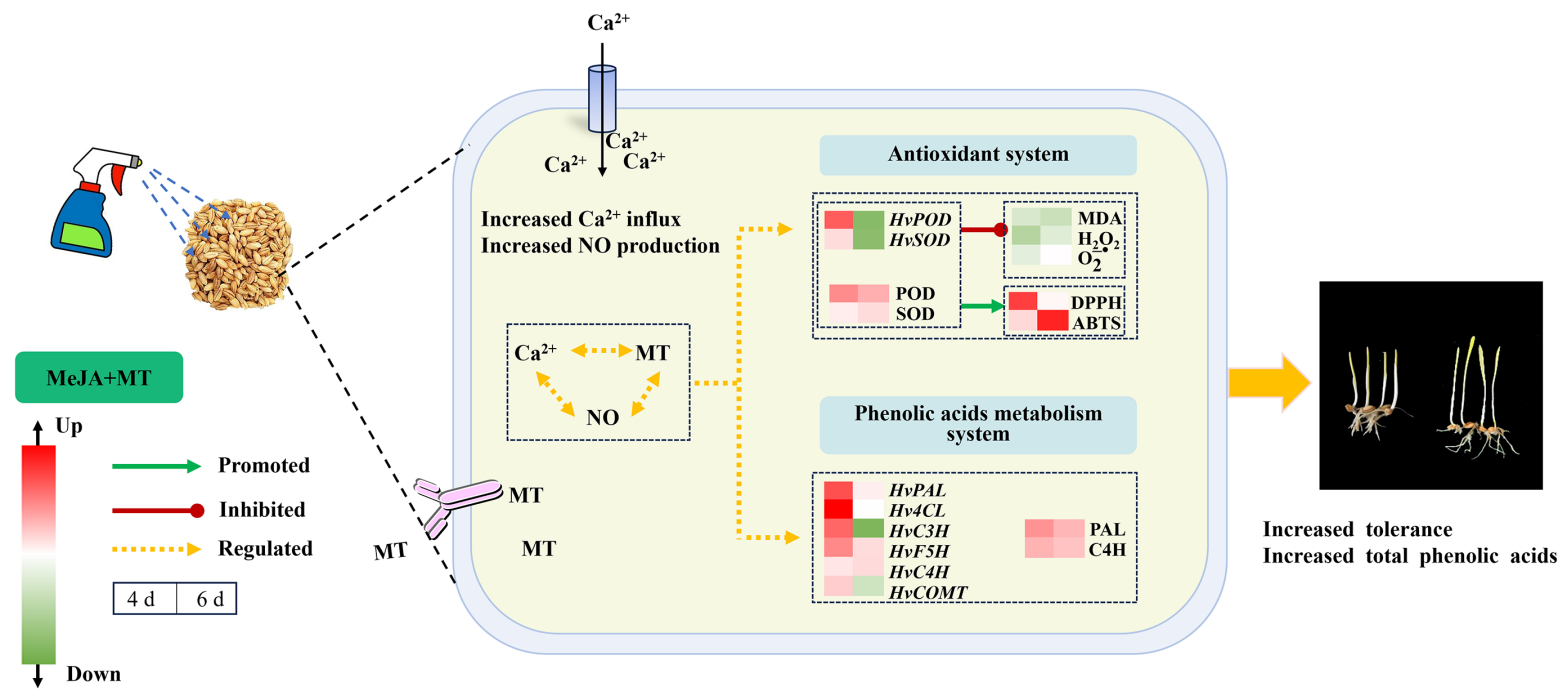
The putative molecular mechanism of MT relieving MeJA stress and stimulating total phenolic acids biosynthesis in barley sprouts.

The findings presented above support the speculative molecular mechanism depicted in Figure 7, illustrating how exogenous MT mitigates the stress induced by MeJA and enhances total phenolic acids biosynthesis in barley sprouts. Exogenous MT (0.1 mM) mitigated the adverse impact of MeJA stress (100 μM) on barley sprouts. This was achieved through enhancing the functionality of antioxidant enzymes, reduced the generation of ROS, and enhanced cell membrane stability. Furthermore, MT was observed to elevate the transcription of genes *HvPAL*, *HvC4H*, *Hv4CL,* and *HvF5H* involved in phenylpropane metabolism, thus promoting the accumulation of phenolic acids. This study provided a reliable material basis for the production of phenolic acid-rich barley sprouts processed products.

## Data availability statement

The datasets presented in this study can be found in online repositories. The names of the repository/repositories and accession number(s) can be found in the article/Supplementary material.

## Author contributions

XT: Writing – original draft. RZ: Data curation, Writing – review & editing. ZY: Validation, Writing – review & editing. JZ: Data curation, Writing – review & editing. WF: Supervision, Writing – review & editing. RY: Writing – review & editing. YY: Writing – review & editing.
