# Supplementary material for: Melatonin mediates phenolic acids accumulation in barley sprouts under MeJA stress
Source: Front Nutr. 2024 Jun 4;11:1403293. doi: 10.3389/fnut.2024.1403293 (PMC11186395; doi:10.3389/fnut.2024.1403293)
Supplement: Supplementary file 1 [file Image_1.pdf]

Supplementary Material

Supplementary Figure

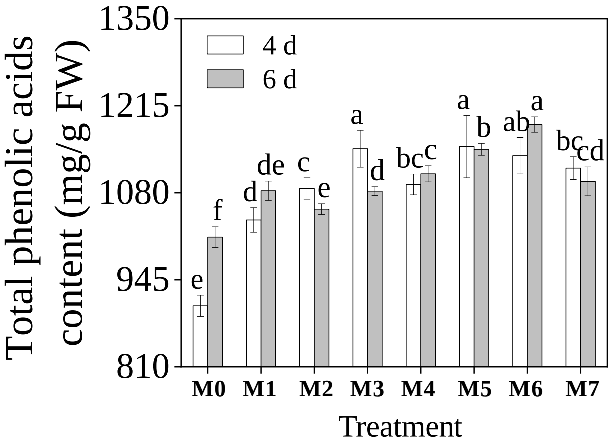

Supplementary Figure 1. Effect of MT concentration on total phenolic acids content of barley sprouts under MeJA treatment. M0: 100 μM MeJA; M1: 100 μM MeJA + 100 μM MT; M2: 100 μM MeJA + 150 μM MT; M3: 100 μM MeJA + 200 μM MT; M4: 100 μM MeJA + 250 μM MT; M5: 100 μM MeJA + 500 μM MT; M6: 100 μM MeJA + 1000 μM MT; M7: 100 μM MeJA + 2000 μM MT.
